# CXCR4 expression in tumor associated cells in blood is prognostic for progression and survival in pancreatic cancer

**DOI:** 10.1371/journal.pone.0264763

**Published:** 2022-03-08

**Authors:** Kirby P. Gardner, Susan Tsai, Mohammed Aldakkak, Stephen Gironda, Daniel L. Adams

**Affiliations:** 1 Creatv MicroTech, Inc., Monmouth Junction, NJ, United States of America; 2 Rutgers University, Graduate School of Biomedical Sciences, Piscataway, NJ, United States of America; 3 The Medical College of Wisconsin Milwaukee, Milwaukee, WI, United States of America; 4 Department of Physiology and Pharmacology, Wake Forest School of Medicine, Winston-Salem, NC, United States of America; Universite de Nantes, FRANCE

## Abstract

The aggressive nature and metastatic potential of pancreatic cancer (PC) results in poor prognosis and high mortality. A better understanding of the underlying biology of PC and the ability of tumor cells to spread to distant sites is needed to advance the treatment of PC. The chemokine receptor CXCR4 has been heavily implicated in the spread and mobility of many solid cancers based on its role in cancer cell chemotaxis as well as increased metastatic potential. To better elucidate CXCR4’s role in the metastatic spread of PC, we examined its expression on various tumor associated cells (TACs) in the peripheral blood of PC patients, including circulating tumor cells (CTCs), epithelial to mesenchymal transition cells (EMTs), and cancer associated macrophage-like cells (CAMLs). In this pilot study, blood samples were procured from 30 PC patients prior to the start of therapeutic intent. CXCR4 expression was analyzed on TACs captured from the blood samples and evaluated in relation to cell migration as well as patient clinical outcomes. In total, CTCs, EMTs, and CAMLs were found in 27%, 60%, and 97% of PC patients, respectively. High CXCR4 expression in CTCs, CAMLs, and EMTs was found to significantly relate to their increased numbers in circulation. Further, higher expression of CXCR4 in CAMLs and EMTs was significantly related to faster progression and worse survival. These data suggest that CXCR4 expression in PC is strongly related to the intravasation and presence of TACs into circulation, as well as being a possible biomarker for aggressive metastatic disease.

## Introduction

Pancreatic cancer (PC) has one of the highest incidences of mortality, ranking third in the United States with an estimated 45,050 deaths in 2020 [1]. PC is projected to rise to the second leading cause of cancer related mortality in the United States by the year 2030 [2]. A partial cause of the high mortality is the metastatic potential of PC, which is initiated via circulating tumor cell’s (CTCs) ability to spread to distant sites [3, 4]. In the “seed and soil” hypothesis, CTCs are theorized to be tumor initiating cells (TICs) that may be targeted to control their spread and metastatic potential [5]. Recently, a second type of tumor associated cell has been discovered, the cancer associated macrophage-like cell (CAML). This cell has been described as a pro-tumorigenic giant myeloid cell found in circulation, along with CTCs in several solid tumor cancers, including PC [6–10]. CAMLs have been shown to reliably predict progression and survival in PC, which may also be related to CTC intravasation into circulation [8–11]. Another circulating tumor associated cell (TAC), that has been implicated in the progression of PC, is the epithelial-mesenchymal transition cell (EMT) [11–13]. EMTs have been examined as a potential biomarker for PC [14]. The intravasation of TICs & TACs are both theorized to aid in the progression and metastasis of various solid tumors, including PC, thus implying the need to better understand, and potentially target receptors involved in the motility of all types of TICs and TACs.

A potential motility receptor that has been implicated in the spread of PC is the C-X-C chemokine receptor type 4 (CXCR4) [15]. CXCR4 has been implicated in the growth of PC, even within premalignant disease such as Pancreatic Intraepithelial Neoplasia (PanIN) [16]. In PC, it has been shown that the secretion of SDF-1, the agonist of CXCR4, from cancer associated fibroblasts aids in the recruitment of pro-tumorigenic myeloid cells to the primary tumor site [17, 18]. As these myeloid cells accumulate in the stroma of PC, they occlude the tumor site from anti-tumorigenic T-cells and immunotherapeutic treatments while simultaneously promoting tumor angiogenesis [15, 19]. Having identified the importance of CXCR4 in the recruitment of myeloid cells to the tumor, the expression of CXCR4 in CAMLs becomes of high interest to study. TICs have shown a strong relation to CXCR4 in PC, with high expressing tumor cells shown to be resistant to chemotherapy treatments, such as gemcitabine [20]. Further, CXCR4 has been implicated in aiding epithelial-to-mesenchymal transition (EMT) [21]. Therefore, examining the expression of CXCR4 on CTCs, EMTs, and CAMLs may provide pertinent prognostic information such as spread, progression, and survival.

As the intravasation of CTCs, CAMLs, & EMTs into circulation has been associated with faster progression and greater metastatic potential, we evaluated the motility receptor CXCR4 expression on these three populations of cells based on their presence in blood, as well as their association with progression free survival (PFS) and overall survival (OS). We established expression ranges of CXCR4 using the model pancreatic cell line PANC-1 via an upregulation with a known activator, the beta-adrenergic receptor agonist isoproterenol [22]. The dual action of the beta-2 adrenergic receptor and the CXCR4 complex modulates G-coupled signaling involved in hematopoietic stem cell trafficking, such as that associated with cancer stem cell invasion [22]. For this prospective study, 30 PC patients who were candidates for surgical resection volunteered a blood sample for CTC, CAML, and EMT analysis. Patients were then tracked through standard of care neoadjuvant therapy and treatment, and then monitored for 24 months to examine the clinical outcomes related to CXCR4 expression on these cell types. Further, CXCR4 expression in CTCs, EMTs, and CAMLs was compared to cell number and patient outcomes to determine if a relationship exists between the motility marker CXCR4 and the three cell types.

## Materials and methods

### Cell culture and bioassay

PANC-1 cells were purchased from ATCC and cultured to confluence according to the ATCC guidelines in DMEM with 10% FBS at 5% CO_2_. Cells were trypsinized and plated overnight on two 8 well plates at 1x10^5 cells/well. After overnight attachment, media was aspirated, and wells from the first plate were treated as follows: A) media alone as an untreated control, B) media+20 μM of isoproterenol for 15 minutes, C) media+20 μM of isoproterenol for 30 minutes, D) media+20 μM of isoproterenol for 60 minutes. The second plate was then treated as follows: A) media alone as an untreated control, B) media+5 μM isoproterenol for 1 hour, C) media+ 10 μM isoproterenol for 1 hour, D) media+ 20 μM isoproterenol for 1 hour, E) media+ 50 μM isoproterenol for 1 hour (All experiments were done in replicates of four). After all experiments, wells were aspirated, and 250 μL of 1% paraformaldehyde in PBS was added to each well for 15 minutes. After 15 minutes, the wells were washed with 1X PBS and replaced with 250 μL of 1% Tween-20 for an additional 15 minutes, followed by 250 μL of blocking in 1XPBS+2% BSA. Wells were washed with 1XPBS and stained as previously described with 1x concentration of CellSieve™ Enumeration Stain Solution (Creatv MicroTech), consisting of FITC conjugated cytokeratin 8, 18, 19 antibody, r-phycoerythrin (PE)-conjugated CXCR4 antibody (Miltenyi), and Cy5-conjugated CD45 antibody for 1 hour. Wells were then washed with 1XPBS+0.1%Tween-20 and mounted using Flouromount-G with DAPI (Southern Biotech).

### Cohort recruitment

A prospective single blind pilot study was initiated to analyze the expression of CXCR4 on CTCs, CAMLs, and EMTs found in the peripheral blood of PC patients. Peripheral blood samples were obtained from 30 PC patients through collaboration with the Medical College of Wisconsin, in accordance with the local institutional review board (IRB) approval and with the patients’ informed written consent. PC patients were recruited from 2012 to 2014, having been referred for pancreatic resection based on initial clinical evaluation. Inclusion criteria required patients with exocrine pancreatic adenocarcinomas above the age of 18. Minors were not permitted in this study. Patients were classified as 37% having resectable (n = 11/30), 40% borderline resectable (n = 12/30), 0% locally advanced (n = 0/30) PC, or 23% patients scheduled for primary resection after previous treatment for metastatic disease (n = 7/30). The cohort had (n = 29) patients with pancreatic ductal adenocarcinoma and (n = 1) patient with acinar adenocarcinoma. Pathological stage was assessed after surgical resection. For this study, pathological stage IV was defined as metastatic at diagnosis, pathologically metastatic based on surgical resection, or expired from advanced disease prior to resection. Prior to the initiation of treatment, 7.5 mL blood samples were procured from patients. The blood samples were collected into CellSave Preservative Tubes (Menarini-Silicon Biosystems), anonymized, and shipped within 96 hours for sample processing to collect TACs. Patients were then monitored for 2 years after baseline blood sample draws with the clinical variables age, gender, resectability, pathological staging if available, neoadjuvant and adjuvant therapy (chemotherapy and radiation therapy), tumor markers (CA19-9 and CEA) and evaluated (Table 1). Patient clinical information was not shared or unblinded until the completion of the study.

**Table 1:** Clinical demographic information for patients.

	(n = 30)
**Age (Median)**	65.5
**Age IQR, Range**	61.25–75.5, 47–90
**Gender**	
**Male**	20(66.6%)
**Female**	10 (33.3%)
**Histology**	
**Pancreatic Adenocarcinoma**	29(96.6%)
**Acinar Adenocarcinoma**	1 (3.4%)
**Pathological Stage**	
**I**	6 (20%)
**II**	8 (27%)
**III**	3 (10%)
**IV**	12 (40%)
**Unknown**	1 (3%)
**Resectability**	
**Resectable**	11 (36.6%)
**Borderline Resectable**	12 (40%)
**Locally Advanced**	0 (0%)
**Metastatic**	7 (23.3%)
**CAMLs Present**	29 (96.7%)
**Median CAML size**	56.5μm (0–189)
**CTCs Present**	8 (26.67%)
**EMTs Present**	18 (60.0%)
**Neoadjuvant Chemo**	26 (86.7%)
**Adjuvant Chemotherapy**	20 (66.7%)
**Radiation Therapy**	16 (53.3%)
**Surgical Resection**	18 (60%)
**Resection Margin**	
**Positive**	1 (3.3%)
**Negative**	17 (56.7%)
**Unknown**	12 (40.0%)

### Tumor associated circulating cell capture

Blood samples were filtered using the CellSieve^TM^ microfiltration system with low-pressure vacuum, as described in previous literature [6–8]. CTCs, EMTs, and CAMLs were evaluated and differentiated as described in previous publications [12]. Briefly, identification of CTCs is based on morphological features, as well as the expression of filamentous cytokeratin 8, 18, and 19, and DAPI, with an absence of CD45. CAMLs were identified based on an enlarged polynucleated nucleus (between 14μm-64μm diameter) and by their enlarged cellular body (roughly between 30μm-300μm in length) positive for cytokeratin and/or CD45 expression. EMTs were identified by their morphology, including their elongated nuclei and lack of CD45 expression [11, 12]. In the assay, 7.5 mL of blood was prefixed for 15 minutes and drawn through a moistified filter at a preset vacuum. After the blood was filtered, microfilters were washed with two rounds of 1 mL of PBS, followed by postfixation, permeabilization, and staining using the antibody solution described above. After the staining step, filters were washed with 10 mL of PBS + 0.1% Tween-20 (PBST), 2 mL of PBS, and mounted onto a glass microscope slide using Fluoromount-G with DAPI (Southern Biotech).

### Analysis of filters

**Fig 1:**
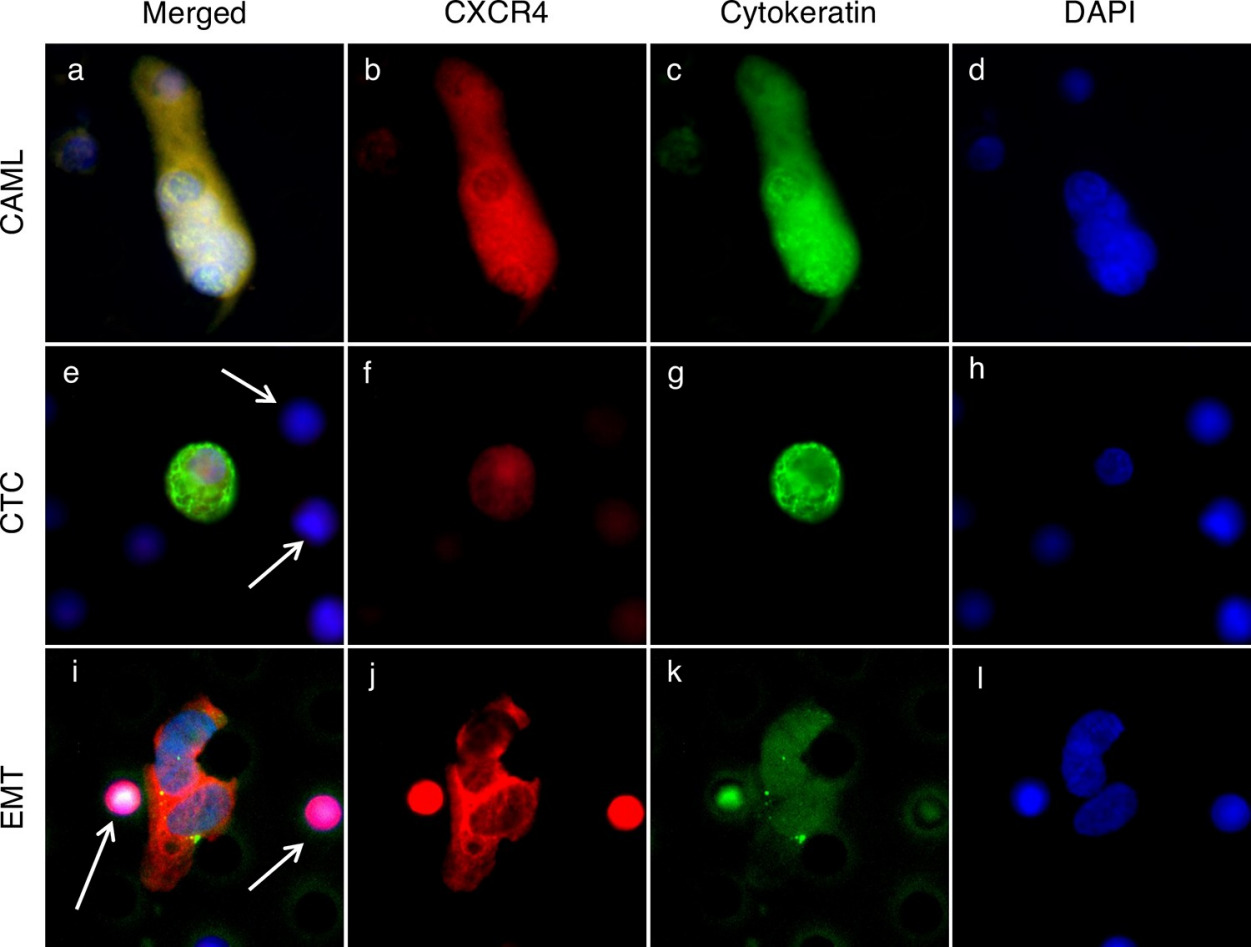
Images of CAML, CTC, EMTs, and white blood cells. **a-d.** CAMLs are enlarged cells with a polyploid nucleus (blue) and diffuse Cytokeratin (green), and can be CXCR4 positive (red). **e-h.** CTCs are cells with a nuclei (blue) and filamented Cytokeratin (green), and can be CXCR4 positive. **i-l.** This is a cluster of EMTs. EMTs are cells typically presenting with smooth, oval shaped nuclei (blue) and weak Cytokeratin (green), and can be CXCR4 positive. White arrows highlight normal white blood cells. Boxes = 60μm.

After samples were filtered and stained according to the protocol listed above, samples underwent CTC, CAML, and EMT enumeration and CXCR4 stain intensity analysis. An Olympus BX51WI fluorescent microscope with a Carl Zeiss AxioCam monochrome camera was used for imaging CTCs, EMTs, and CAMLs (Fig 1). The Zen2011 Blue program was used to process the images. Cell size was measured using the precalibrated size tools in the Zen2011 Blue software.

CAMLs are enlarged cells with a polyploid nucleus (blue) and diffuse Cytokeratin (green) and can be CXCR4 positive (red) (Fig 1A–1D). CTCs are cells with a nuclei (blue), filamented Cytokeratin (green), and can be CXCR4 positive (Fig 1E–1H). EMTs are cells typically presenting with smooth, oval shaped nuclei (blue) and weak Cytokeratin (green), and can be CXCR4 positive (Fig 1I–1L). White arrows highlight normal white blood cells. Boxes = 60μm.

### Statistical analysis

Cox proportional hazard regression were used in this manuscript to determine the univariate analysis of the cells at a statistical analysis’s threshold of p≤0.05, using MATLAB R2020. Wilcoxon ranked sum tests were used to determine p-values in comparing CAML numbers, EMT numbers, and CTC numbers based on CXCR4 expression (Fig 1). Progression free survival (PFS) and overall survival (OS) Kaplan-Meier estimation was done using the time to progression, defined as the interval between when the first blood sample was obtained to the date of progression, by standard RECIST criteria using PET/CT scan or death, within 24-month end point.

## Results

**Fig 2:**
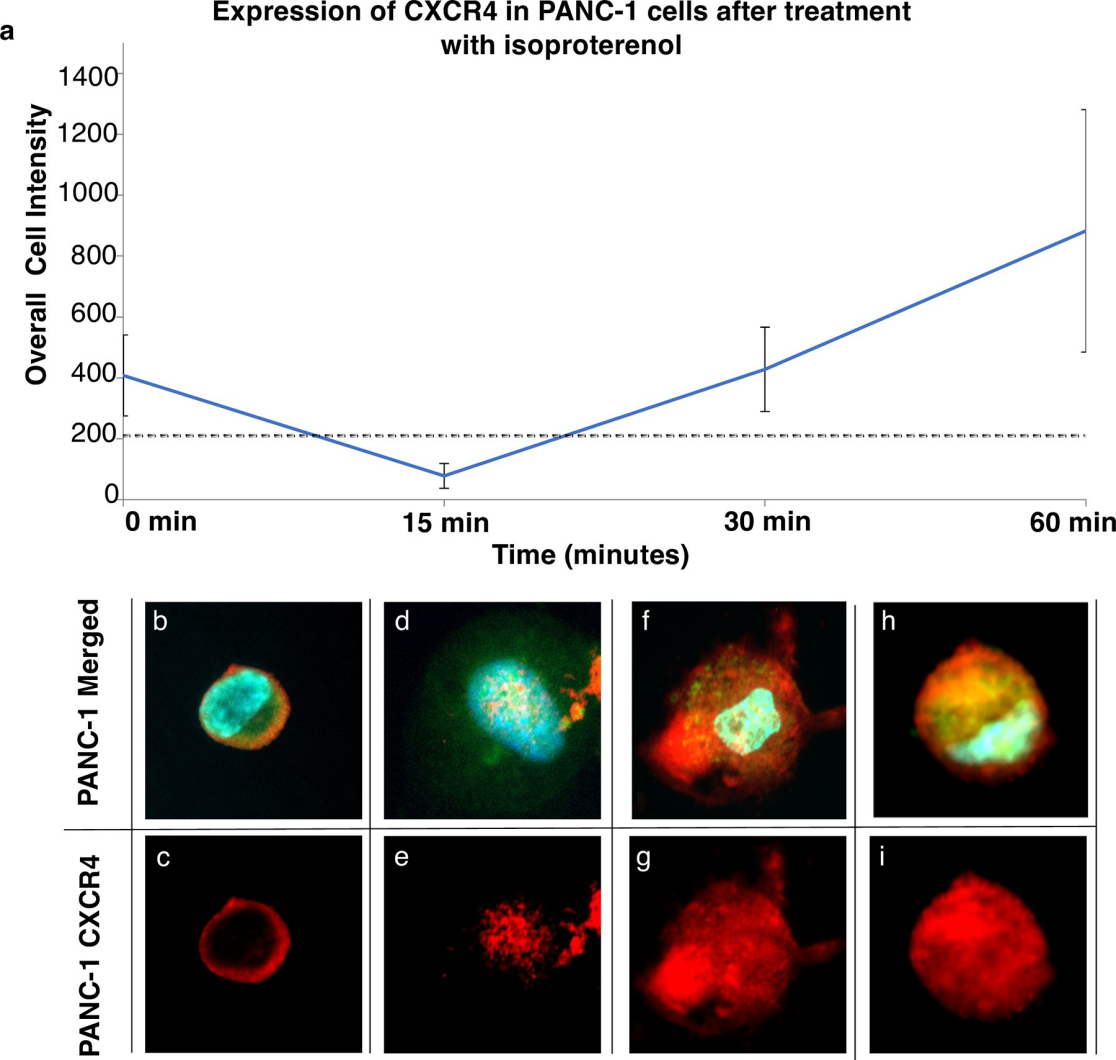
CXCR4 intensity changes, internalization and upregulation in PANC-1 cells after exposure to isoproterenol. **a.** Intensities of CXCR4 in PANC-1 cell lines compared to exposure times using 20μm isoproterenol. Black dotted line ~2x standard deviation (Intensity = 210). Error bars = Standard Error. **b & c.** Inactive CXCR4 is localized to the perimeter of the cell surface. **d & e.** In presence of 15 min of isoproterenol, CXCR4 internalizes into endocytic compartments within the cells, causing a lowering of overall signal intensity. **f & g.** After 30 min, the CXCR4 can be seen within endocytic compartments, on the cell surface, and with increasing signal, as CXCR4 begins recycling to the surface. **h & i.** Cells increase CXCR4 production in response to high stimuli. **b, d, f & h.** Merged cell images with DAPI (blue), cytoplasm stain (green), and CXCR4 (red). Boxes = 45 μm.

CXCR4 expression profiles were initially modeled in PANC-1 cell lines, both before and after treatment with the beta-adrenergic receptor agonist isoproterenol (Fig 2 and S1 Fig). Average CXCR4 expression for PANC-1 cells was measured prior to treatment and 15 minutes, 30 minutes, and 60 minutes after treatment with isoproterenol (Fig 2A). Prior to isoproterenol treatment, expression of CXCR4 in PANC-1 model pancreatic cell lines appeared as a general surface stain when grown in standard media (Fig 2B and 2C) with an average cell intensity of ~408 (Fig 2A). When PANC-1 cells were exposed to isoproterenol for 15 minutes, it was observed that CXCR4 expression dropped to an average intensity of 78 (Fig 2A), with much of the signal appearing localized to the nucleus of the cell (Fig 2D and 2E). After continued exposure to isoproterenol for 30 min, it was observed that CXCR4 expression appeared to increase to its pre-activation levels of an intensity of 428 (Fig 2A). This signal stabilization appeared to result from the CXCR4 re-emerging on the cell surface (Fig 2F and 2G). Upon further exposure with isoproterenol treatment (60 min), CXCR4 expression then continued to increase, with a ~200% increase in intensity to 883 after 1 hour exposure (Fig 2A), caused by increased CXCR4 signal both within the cell and on the cell surface (Fig 2H and 2I), This result is in line with previous publications, and the general understanding of CXCR4 activation in model breast cancer cell lines (e.g. MB231 cells), which has shown that activation of CXCR4 results in an initial internalization of CXCR4 to the nuclear area [23], with further internalization in the presence of very high concentrations of isoproterenol (S1 Fig). In line with prior publications, over time, continued activation results both in the CXCR4 recycling back to the cell surface and in the upregulation of expression in response to certain stimuli. Here, we used these findings to differentiate CXCR4 expression level in inactivated versus activated pancreatic cells to build a theoretical model expression system to evaluate low versus high CXCR4. In PANC-1 cells it appeared that a signal of 2 times the standard deviation at 15 minutes (~210) is the threshold between low/no expression of CXCR4 versus high expression (Fig 2A).

We recruited a pilot study of 30 blood samples from PC patients diagnosed with pancreatic adenocarcinoma to screen with the CXCR4 assay model (Table 1). The mean age of the cohort was 67.4 years old with an age range of 47 to 90 years and an interquartile range of 61.25 to 75.5 years. At the clinical diagnosis, CT scans were used to assign resectability of patients. Pathological assessment of the cohort was 20% (n = 6) were stage pI, 27% (n = 8) were stage pII, 10% (n = 3) were stage pIII, and 40% (n = 12) were stage pIV, and 3% (n = 1) did not have pathological staging due to no surgical resection or dropped off study (Table 1). At the end of 24 months, 33% (n = 10/30) did not progress, with CAMLs, EMTs, and CTCs found in 97%, 60%, and 27% of PC patients respectively. Within the metastatic population, all patients had CAMLs present, 67% had EMTs present, and 42% had CTCs present. Of all TACs captured, CTCs made up 2% (n = 23/1066) of TACs, EMTs made up 74% (n = 793/1066), and CAMLs made up 23% (n = 250/1066).

**Fig 3:**
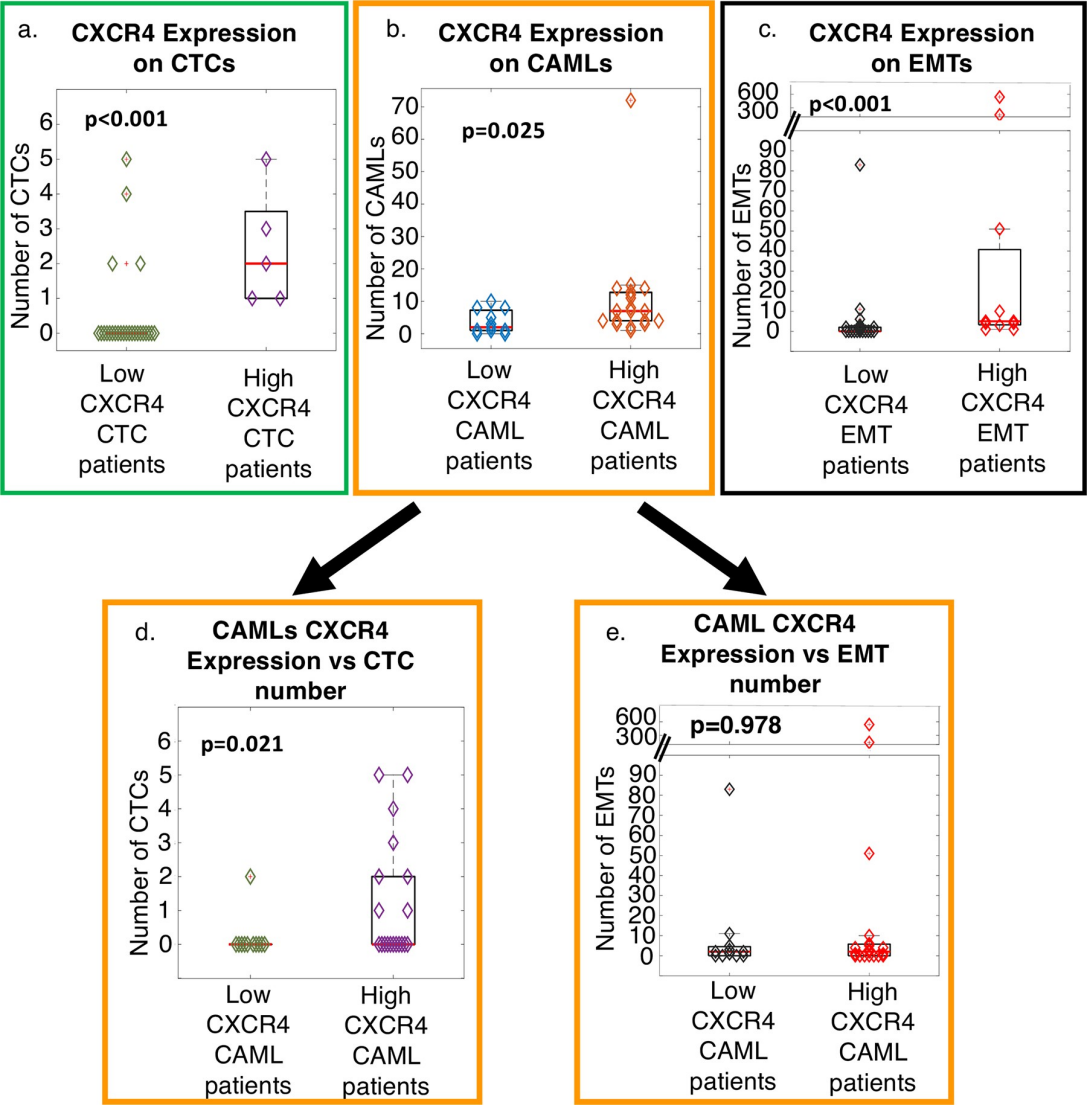
Relationship of CXCR4 expression and number of CTCs, CAMLs, and EMTs in circulation. **a.** Whisker Plots of patient’s CTC number based on high/low expression of CXCR4. Wilcoxon ranked sum (p<0.001) **b.** Whisker Plots of patient’s CAML number based on high/low expression of CXCR4. Wilcoxon ranked sum (p = 0.025). **c.** Whisker Plots of patient’s EMT number based on high/low expression of CXCR4. Wilcoxon ranked sum (p<0.001). **d.** Relationship of patient’s CTCs number based on expression of CAML CXCR4. Wilcoxon ranked sum (p = 0.021) **e.** Relationship of patient’s EMT number based on expression of CAMLs CXCR4. Wilcoxon ranked sum was nonsignificant (p = 0.978).

In the various populations of TICs/TACs, CXCR4 expression was examined to evaluate if a relationship exists between the aggressiveness of the tumor and their numbers in the blood stream. High CXCR4 expression in CTCs appear to significantly relate to high numbers of CTCs in circulation (p<0.001) (Fig 3A). High CXCR4 expression in CAMLs also appeared to significantly relate to the presence of more CAMLs in circulation (p = 0.025) (Fig 3B). Additionally, high CXCR4 expression in EMTs significantly related to more EMTs in circulation (p<0.001) (Fig 3C). Interestingly, upon further analysis of the 3 cell populations, a relationship was found between CXCR4 in CAMLs and CTCs. Specifically, patients with higher expression of CXCR4 in CAMLs were found to have more CTCs in circulation (p = 0.021) (Fig 3D), but this relationship did not exist for number of EMTs (p = 0.978) (Fig 3E). When comparing all relationships between the CXCR4 on CTCs or EMTs, no relationship was found with number of CAMLs (S2 Fig).

**Fig 4:**
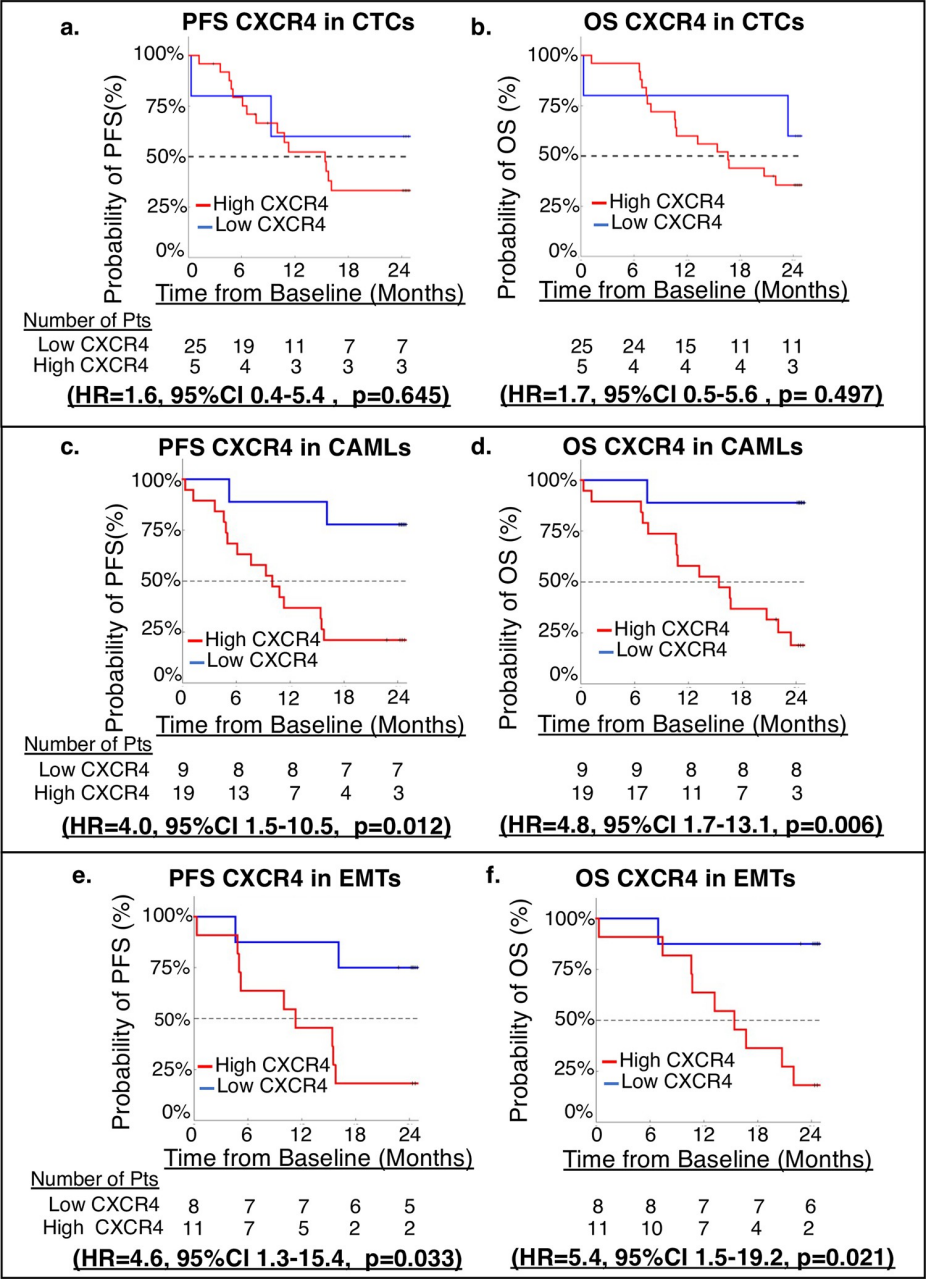
Kaplan-Meier graphs of PFS and OS for CTCs, CAMLs, and EMT expression. **a.** PFS of CTC CXCR4 Expression. **b.** OS of CTC CXCR4 Expression. **c.** PFS of CAML CXCR4 Expression. **d.** OS of CAML CXCR4 Expression. **e.** PFS of EMT CXCR4 Expression. **f.** OS of EMT CXCR4 Expression.

After identifying a relationship between CXCR4 and cells in circulation, CXCR4 expression was analyzed to see the effect compared with patients’ rate of progression and survival. CXCR4 in CTCs was not found to be a significant predictor of progression (HR = 1.6, 95%CI 0.4–5.4, p = 0.645) (Fig 4A) nor OS (HR = 1.7, 95%CI 0.5–5.6, p = 0.497) (Fig 4B). However, higher CXCR4 expression in CAMLs was related to significantly faster progression (HR = 4.0, 95%CI 1.5–10.5, p = 0.012) (Fig 4C) and significantly higher mortality (HR = 4.8, 95%CI 1.7–13.1, p = 0.006) (Fig 4D). In addition, expression of CXCR4 on EMTs also appear to relate to progression and survival, with patients with high CXCR4 expressing EMTs having significantly faster progression (HR = 4.6, 95%CI 1.3–15.4, p = 0.033) (Fig 4E) and higher mortality rate (HR = 5.4, 95%CI 1.5–19.2, p = 0.021) (Fig 4F).

## Conclusion

In this work, we evaluated the pancreatic cancer model cell line PANC-1for activation, internalization, and upregulation of CXCR4 following exposure to an activator, Isoproterenol. We then built a model expression profile of CXCR4 based on low expression or high expression of CXCR4 signal in PANC-1 cells. We used these CXCR4 expression profiles to help establish a threshold to later be used in patient analysis. We then evaluated this profile in three circulating cell populations known to originate from primary tumors using 30 PC patients comparing both presence of the TAC populations and their effect on patient outcome.

We began by running a model bioassay using the established CXCR4 activator, isoproterenol, to induce changes in CXCR4 expression within PANC-1 cells. It was observed that after exposure to the isoproterenol agonist, both internalization and recycling of the CXCR4 could be seen. Interestingly, this internalization and subsequent recycling of CXCR4 receptor has been demonstrated in other solid cancer types [23]. The highest level of CXCR4 expression occurred at one-hour post exposure to isoproterenol at 20 μM which has been observed in previous works on the stimulation of cancer cells with beta agonists [22, 23]. The suggested pattern based on this assay is that initial exposure to isoproterenol (Fig 2C) causes a subsequent theoretical co-internalization with beta-adrenergic receptors (Fig 2E), followed by CXCR4 recycling to the cell surface (Fig 2G) and upregulation (Fig 2I) of CXCR4 receptors. We established the threshold of CXCR4 signal to be 210.

We evaluated CXCR4 expression on cells which had intravasated into blood circulation from tumors from the 30 PC patients. It was found that higher CXCR4 expression on CTCs and EMTs was related to a greater number of CTCs and EMTs in circulation respectively (Fig 3A & 3C). Further, higher expression of CXCR4 on CAMLs not only led to more CAMLs in circulation, but there was a relationship between CAML CXCR4 and increased presence of CTCs in the blood. The relationship between CTCs and CAMLs in circulation based on CXCR4 in CAMLs was a surprising finding and may suggest a possible migratory relationship between CAMLs and CTCs. This association could suggest that CAMLs may be aiding in the intravasation of CTCs, and that CXCR4 may act as the motility axis. Interestingly, Adams et al. (2014) had initially suggested a tandem intravasation in a patient’s blood with tumor cells upon observing CAMLs and CTCs bound together in circulation [8]. Here we find further evidence of this hypothesis indicating that higher expression of the motility marker CXCR4 on CAMLs appears to relate to an increase of all tumor associated cells in circulation, including CTCs. CXCR4 expression may aid in the hypothetic relationship between CXCR5 expression in macrophages and the spread of CTCs. However, this relationship between CXCR4 expression in CAMLs and CTCs in circulation was the only significant relationship found between the three cell types. Hopefully future studies can help better elucidate these cells interplay in the tumor microenvironment and intravasation process.

CXCR4 has been implicated in the migration of hematopoietic stem cells and myeloid cells [18]. CAMLs express both myeloid lineage and hematopoietic expression profiles, based on their positivity for CD14, CD45, and CD133 [6]. Therefore, it is possible to theorize that patients with higher expression of CXCR4 on CAMLs (i.e. hematopoietic myeloid cell) would have more CAMLs found in circulation based on CXCR4 involvement in recruiting myeloid cells to the tumor site. Further, it has been demonstrated that having an abundance of myeloid cells in the stroma of PC prevents phagocytic T-cells from infiltrating the tumor as well as preventing therapies from reaching the tumor [15]. If these CAMLs behave similar to M2 macrophages, they may be occluding the tumor from receiving anti-tumorigenic effects of T-cells and therapies, though the pro-tumorigenic relationship between CAMLs and the primary PC needs to be further elucidated.

In this study, high expression of CXCR4 on CAMLs and EMTs was prognostic of progression and worsened overall survival of PC patients. This suggests that CAMLs may be an excellent prognostic tool in PC and appear to coincide with previous publications on CAMLs in PC as it relates to progression and survival [9, 10, 24]. As high CXCR4 expression in CAMLs appeared to correspond with more CAMLs in circulation (Fig 3B), and previous publications on CAMLs in PC found that higher numbers of CAMLs in circulation was predictive of progression [9]. The data in this study suggests that high expressions of CXCR4 in CAMLs can also stratify progression and survival (Fig 4C and 4D). It is reasonable that further studies need to be conducted to analyze the potential relationship between CXCR4 expression on CAMLs, CAML number, and progression in PC.

CXCR4 aids in EMT transition and PC cell invasion [21]. The transition from epithelial to mesenchymal phenotypes increases migratory and invasive properties and promotes metastasis [25]. The increase in expression of CXCR4 on EMTs is an interesting finding and may indicate that increases in invasiveness based on higher CXCR4 expression leads to higher numbers of cells in circulation (Fig 3C). This would further implicate EMT PC cell invasion by their significant relationship to progression and survival (Fig 4E and 4F). Although high CXCR4 expression on CTCs appears to correlate with greater CTCs in circulation, expression was not a significant predictor of progression or survival (Fig 4A and 4B). Our findings show that EMTs may prove to be useful as a potential prognostic biomarker for treatment options, though the relationship between CXCR4 expression, EMT numbers, progression, and overall survival needs to be explored in more detail.

In this initial pilot study, it appears that expression of CXCR4 in CAMLs may predict progression and overall survival in PC, and has a relationship to the presence of CTCs entering circulation. While there is a clear need to expand this study and validate these preliminary findings, it would be of interest to measure CXCR4 in TACs of patients undergoing anti-CXCR4 targetable therapies, such as clinical trials NCT02907099 and NCT04177810. Sequential monitoring of CXCR4 as a predictive biomarker during therapeutic intent [17] may provide information on patients who are likely to benefit from CXCR4 therapy, or patients which might become resistant to CXCR4 therapies during treatment. Further, there is potential to expand upon these initial findings by co-staining CAMLs, CTCs, and EMTs with anti-PD-L1 fluorescent antibodies, which has been shown by a number of groups to predict patient response to anti-PD-L1/PD-1 therapies [26, 27]. Several current clinical trials include anti-PD-L1 therapies alongside anti-CXCR4 therapies in PC (i.e. trials NCT02907099 and NCT04177810). By quantifying expression of both PD-L1 and CXCR4 together on CAMLs, CTCs, and EMTs, we might be able to interpret which patients may benefit from the conjunction of these two therapies. Regardless, the role of CXCR4 appears to have a very important role in the progression and spread of PC, thus a better understanding of expression’s influence on TACs could help direct clinical decisions and the usefulness of immunotherapy in newly diagnosed PC.

## Supporting information

S1 Fig CXCR4 intensity and endosome formation after increasing concentrations of isoproterenol. **a.** Intensity of CXCR4 in PANC-1 cell line compared to increasing concentration of isoproterenol. **b-f.** Merged images of PANC-1 cells exposed to isoproterenol at 0, 5, 10, 20, and 50 μM for 60 minutes. Nucleus (light Blue), Cytoplasm (green), CXCR4 (red). **g-k.** CXCR4 images of PANC-1 cells. More endosome formation (see as intense dots) occurs closer to the perinuclear space at increased concentrations of isoproterenol. Optimal CXCR4 upregulation occurred with 20μM, with 50μM showing lower expression caused by toxicity to the cells. Boxes = 45 μm.
(PDF)

S2 Fig CXCR4 expression on CTCs vs number of CAMLs and EMTs & CXCR4 expression on EMTs vs number of CAMLs and CTCs. **a.** Whisker plots of average CXCR4 expression on CTCs compared to number of CAMLs. (Wilcoxon ranked sum test p = 0.542) **b.** Whisker plots of average CXCR4 expression on CTCs compared to number of EMTs. (Wilcoxon ranked sum test p = 0.604) **c.** Whisker plots of average CXCR4 expression compared to number of CAMLs. (Wilcoxon ranked sum test p = 0.289). **d.** Whisker plots of average CXCR4 expression on EMTs compared to number of CTCs (Wilcoxon ranked sum test p = 0.440).
(PDF)
