# Supplementary material for: CXCR4 expression in tumor associated cells in blood is prognostic for progression and survival in pancreatic cancer
Source: PLoS One. 2022 Mar 8;17(3):e0264763. doi: 10.1371/journal.pone.0264763 (PMC8903256; doi:10.1371/journal.pone.0264763)
Supplement: S1 Fig — a. Intensity of CXCR4 in PANC-1 cell line compared to increasing concentration of isoproterenol. b-f. Merged images of PANC-1 cells exposed to isoproterenol at 0, 5, 10, 20, and 50 μM for 60 minutes. Nucleus (light Blue), Cytoplasm (green), CXCR4 (red). g-k. CXCR4 images of PANC-1 cells. More endosome formation (see as intense dots) occurs closer to the perinuclear space at increased concentrations of isoproterenol. Optimal CXCR4 upregulation occurred with 20μM, with 50μM showing lower expression caused by toxicity to the cells. Boxes = 45 μm. (PDF) [file pone.0264763.s001.pdf]

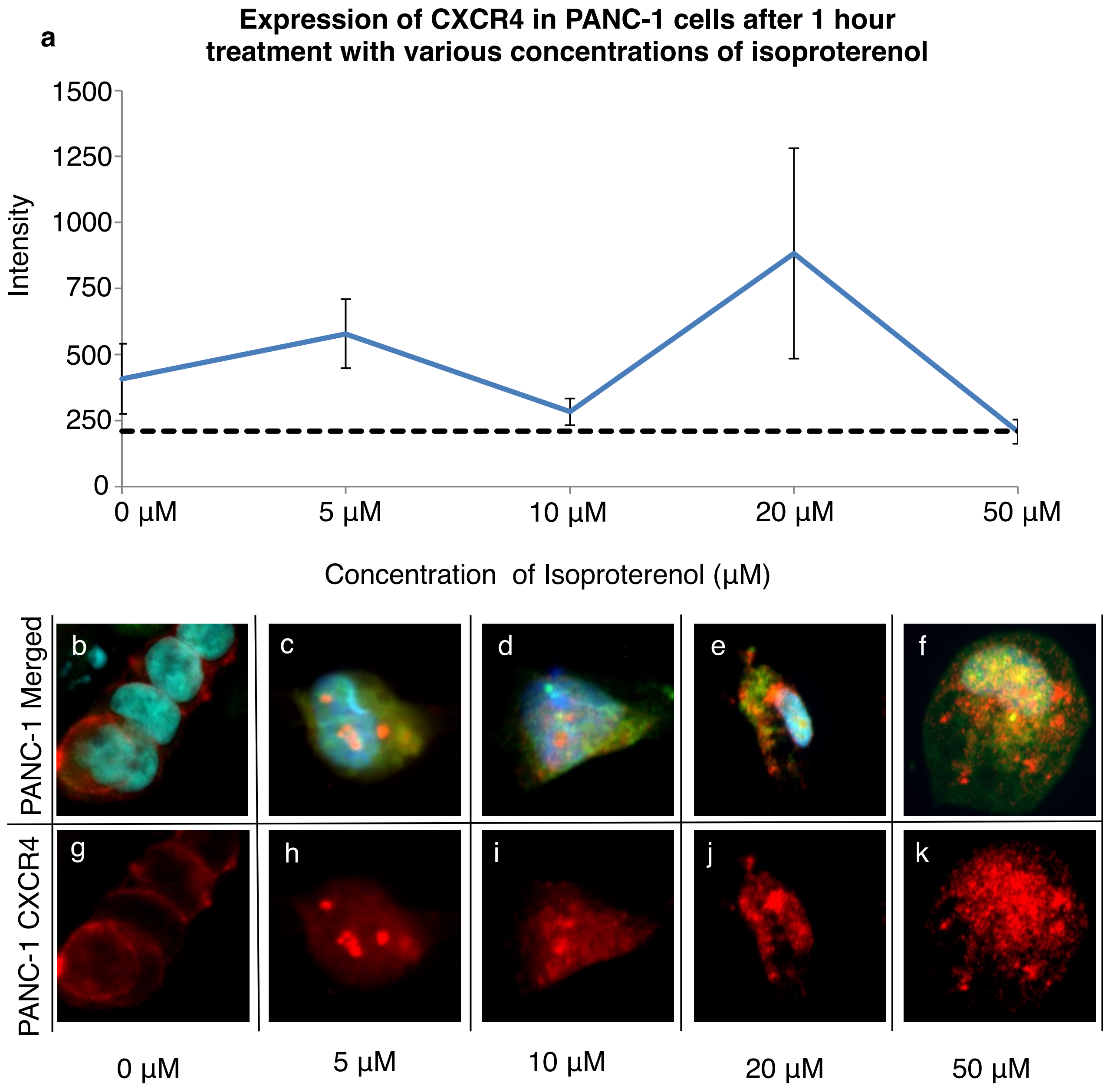
a
Expression of CXCR4 in PANC-1 cells after 1 hour treatment with various concentrations of isoproterenol
Intensity
1500
1250
1000
750
500
250
0
0 µM
5 µM
10 µM
20 µM
50 µM
Concentration of Isoproterenol (µM)
PANC-1 Merged
PANC-1 CXCR4
b
c
d
e
f
g
h
i
j
k
0 µM
5 µM
10 µM
20 µM
50 µM
