# Supplementary figures and images for: CXCR4 expression in tumor associated cells in blood is prognostic for progression and survival in pancreatic cancer
Source: PLoS One. 2022 Mar 8;17(3):e0264763. doi: 10.1371/journal.pone.0264763 (PMC8903256; doi:10.1371/journal.pone.0264763)

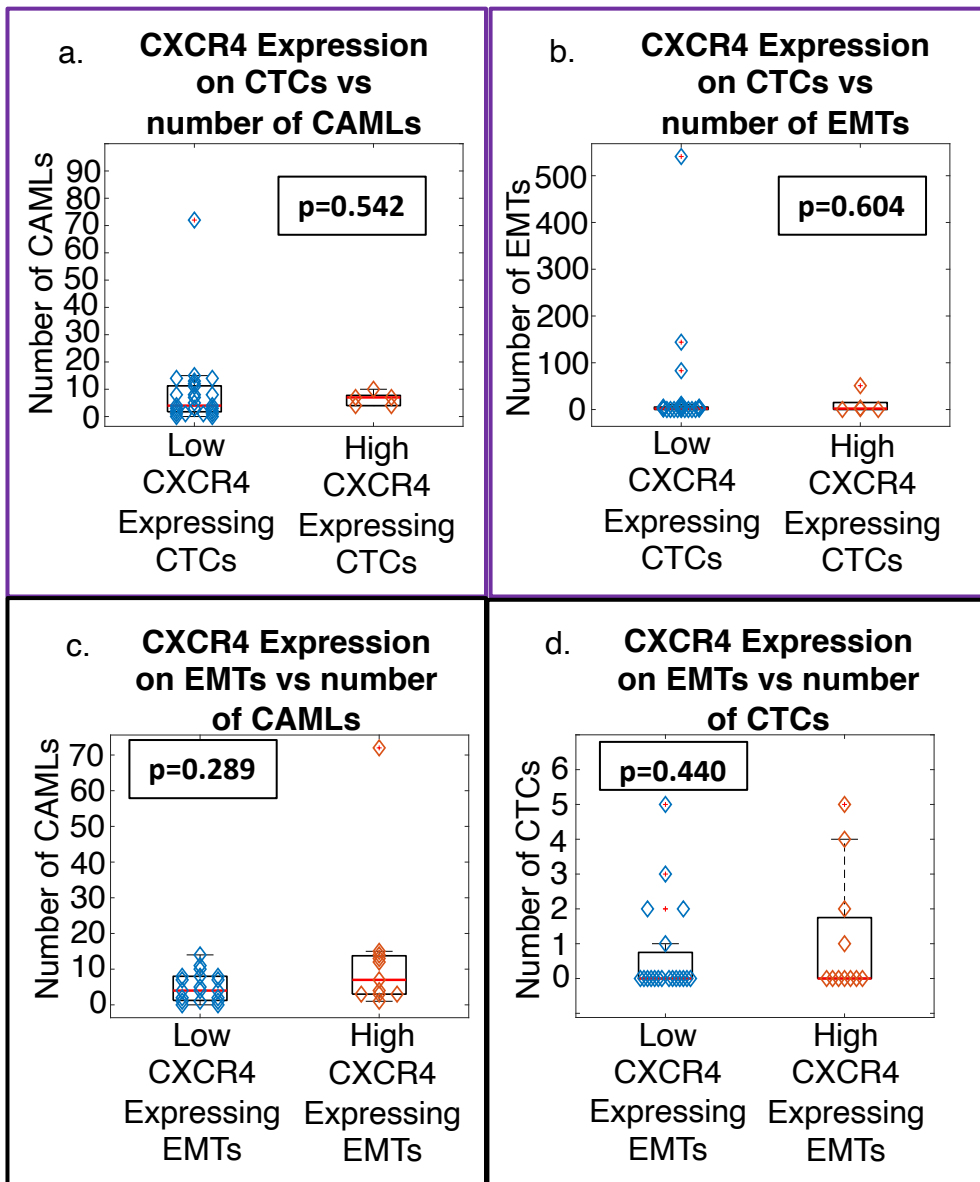

Supplement: S2 Fig — a. Whisker plots of average CXCR4 expression on CTCs compared to number of CAMLs. (Wilcoxon ranked sum test p = 0.542) b. Whisker plots of average CXCR4 expression on CTCs compared to number of EMTs. (Wilcoxon ranked sum test p = 0.604) c. Whisker plots of average CXCR4 expression compared to number of CAMLs. (Wilcoxon ranked sum test p = 0.289). d. Whisker plots of average CXCR4 expression on EMTs compared to number of CTCs (Wilcoxon ranked sum test p = 0.440). (PDF) [file pone.0264763.s002.pdf]
